# Whole-Genome Sequence of *Enteractinococcus helveticum* sp. nov. Strain UASWS1574 Isolated from Industrial Used Waters

**DOI:** 10.1128/genomeA.00756-16

**Published:** 2016-07-28

**Authors:** Julien Crovadore, Gautier Calmin, Romain Chablais, Bastien Cochard, François Lefort

**Affiliations:** a Plants and Pathogens Group, Research Institute Land Nature and Environment, hepia, HES-SO University of Applied Sciences and Arts Western Switzerland, Geneva, Switzerland; b Faculty of Engineering and Architecture, HES-SO University of Applied Sciences and Arts Western Switzerland, Delémont, Switzerland

## Abstract

We report here the whole-genome shotgun sequences of the strain UASWS1574 of the undescribed *Enteractinococcus helveticum* sp. nov., isolated from used water. This is the first genome registered for the whole genus.

## GENOME ANNOUNCEMENT

*Enteractinococcus* is a new Gram-positive bacterial genus of the *Micrococcaceae* family created in 2012 (1) that contains four described species, *E. coprophilus* Cao et al. 2012*, E. fodinae* Cao et al. 2012 (formerly *Yaniella fodinae* Dhanjal et al. 2011), *Enteractinococcus lamae* Chen et al. 2015 (strain YIM 101617), and *E. viverrae* Chen et al. 2015 (strain YIM 101632) (2), as well as three yet-undescribed species, including this strain. Mostly isolated from animal feces and soil, these bacteria are aerobic and nonmotile, coccoid to oval (0.5 to 1.5 µm diameter), and occur singly or in clusters. Growth was observed at 25 to 40°C (optimum 28°C) and at pH 7.0 to 11.0 (optimum pH 8.0); these species have a GC content in the range of 55.9 to 61.6% (1, 2). 

 The strain UASWS1574 was isolated from aerobic granules of industrial sewage sludge in an experiment of selection for highly ammonia-tolerant nitrifying bacteria. It was initially identified as belonging to the genus *Enteractinococcus* by 16S sequencing because it displayed 96 to 98% identity with the four known *Enteractinococcus* spp. Genomic DNA was extracted from a pure axenic culture grown to stationary phase following an adapted protocol (3). Libraries were generated using the TruSeq Nano DNA LT library kit (Illumina, USA). Whole-genome shotgun sequencing was carried out within one Illumina MiSeq run with 2 × 250-bp paired-end read lengths, using the MiSeq reagent kit version 2 (Illumina) and providing a 114× genome coverage. Trimming and quality-control of the reads were performed with FastQC (4). Genome assembly was computed with SPAdes Genome assembler version 3.7.1 (5). The resulting contigs were arranged with BioEdit (6) and analyzed with QUAST (7). The final assembly yielded 118 contigs (≥200 bp.) with a total genome length of 3,670,653 bp, a GC content of 56.29%, and an *N*_50_ value of 176,870 bp. 

Automated gene annotation was carried out by the NCBI Prokaryotic Genome Automatic Annotation Pipeline (PGAAP) (8) and reviewed with RAST version 2.0 (9). PlasmidFinder (10) did not detect any plasmid, which was confirmed by the RAST and PGAAP analyses. This bacterium owned 3,448 protein-coding sequences (CDSs) distributed in 362 subsystems, in which PGAAP identified 3,427 genes for 3,364 CDSs and 3,201 coding genes, 163 pseudogenes, and 63 RNA genes (5S, 16S, 23S, tRNAs, and ncRNAs). No complete transposon or phages were found integrated. The annotation confirmed the absence of toxins and superantigens, and virulence and disease genes were absent, therefore allowing this bacterium to be considered for industrial and environmental uses. The bacterium is equipped with resistance genes against metals such as arsenic, cadmium, chrome, cobalt, copper, mercury, and zinc and against a few antibiotics (penicillin, fluoroquinolones, and vancomycin). The bacterium is fully equipped for nitrate and nitrite ammonification, ammonia assimilation, and denitrification. With 60 genes involved in the metabolism of isoprenoids, this bacterium could be of interest for industry. One gene encodes a cyclohexene synthase, and two other enzymes are involved in cyclohexane and cyclohexanone degradation. Additionally, 68 genes are active in a wide variety of degradation pathways of aromatic compounds, thus offering a possible role in depollution of wastewater and contaminated soils.

### Nucleotide sequence accession numbers.

This whole-genome shotgun project was deposited at DDBJ/EMBL/GenBank under the accession number LXEY00000000. The version described in this paper is the first version, LXEY00000000.1. The 118 contigs have been deposited under the accession numbers LXEY01000001 to LXEY01000118.
